# Progress in preventive therapy for cancer: a reminiscence and personal viewpoint

**DOI:** 10.1038/s41416-018-0039-4

**Published:** 2018-04-23

**Authors:** Jack Cuzick

**Affiliations:** Centre for Cancer Prevention, Wolfson Institute of Preventive Medicine, Queen Mary University of London, Charterhouse Square, London, EC1M 6BQ UK

## Abstract

Prophylactic drug treatment with aspirin, statins and anti-hypertensive agents has had a major impact on the incidence of cardiovascular disease and is now well established. Progress in therapeutic cancer prevention has been much slower; only recently have effective agents been clearly established. Breast cancer has led the way and endocrine agents used to treat it—notably tamoxifen and the aromatase inhibitors—have now been shown to have a substantial preventive effect as well. However, these agents carry some toxicity and thus identifying high-risk women who are likely to benefit most is a key priority. In contrast, the ability of low-dose aspirin to prevent about one-third of colorectal, gastric, and oesophageal cancers, combined with its much lower toxicity profile, make it attractive for a much larger proportion of the general population. Vaccination against the human papilloma virus is also a preventive intervention with large benefits for the whole population. Here I recall my involvement in these initiatives and offer a personal viewpoint on what has been achieved and what remains to be done.

## Breast Cancer Prevention

Breast cancer has led the way in terms of developing preventive therapy for cancer. My interest began in 1979 when I first joined the Imperial Cancer Research Fund. I came to London from Oxford, to work on the Guernsey study of hormonal risk factors for breast cancer with Mick Bulbrook and John Hayward. Oestrogen had been linked, in some not fully understood way, as a major risk factor and the earliest observations go back over 100 years to Beatson,^1^ who reported that oophorectomy reduced recurrence rates in women with breast cancer. Increased oestrogen levels such as those associated with oral contraceptives (IARC, 1979),^2^ postmenopausal hormone therapy^3,4^ and postmenopausal obesity^5^ had been shown to increase breast cancer risk. However, little real progress was made in showing that reducing the oestrogen stimulus could reduce risk until Jordan^6^ reported that tamoxifen treatment led to lower breast cancer rates in 7,12-Dimethylbenz(a)anthracene (DMBA)-induced tumours in rats.

**Fig. 1:**
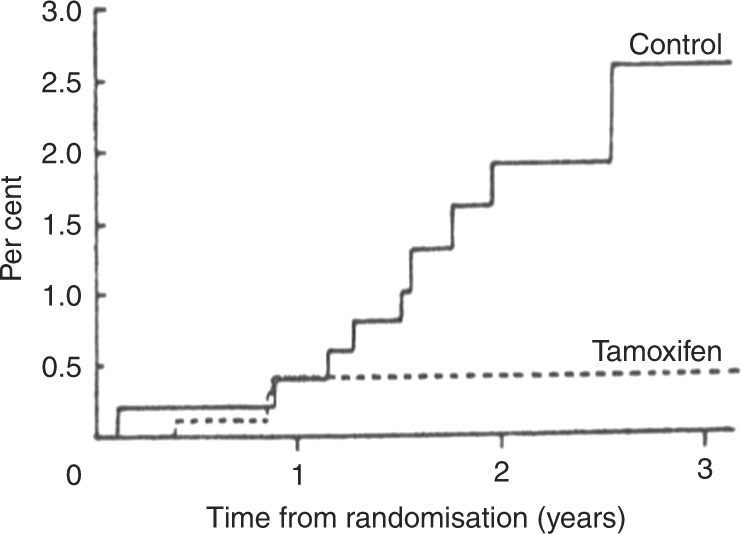
Initial data showing tamoxifen prevented contralateral tumours in an adjuvant trial of tamoxifen vs placebo (Reprinted with permission from ref.^6^)

The first human evidence came in trials using tamoxifen for the treatment of breast cancer, where reductions in new contralateral tumours were first reported in 1985^7^ (Fig. 1). This was followed by a range of confirmatory reports in other adjuvant trials, summarised by the Early Breast Cancer Trials Coordinating Group overview.^8^ A comprehensive justification for the use of tamoxifen in cancer prevention was provided in 1986^9^ and, following a lively meeting of UK breast cancer specialists, Trevor Powles was the first to take up this proposal and he conducted a pilot study of tamoxifen in high-risk women at the Royal Marsden Hospital. Initially this was a small study of 200 women, to investigate toxicity and acceptability; however, concern about liver cancer in rats given high doses of tamoxifen and a general conservativeness about the concept of cancer prevention delayed major trials for another 6 years.

Meanwhile, Powles’ study successively obtained permission to increase sample size and eventually it became a study of 2500 women—the largest ‘pilot study’ in history to my knowledge. Finally, in 1992, three large national and international studies began recruitment—our International Breast Cancer Intervention Study-1 (IBIS-I) study in the UK, Australia and New Zealand; a North American study—NSABP-P1, coordinated by the National Surgical Adjuvant Breast and Bowel Project; and a study in hysterectomised women in Italy (Table 1). The IBIS-I study was truly a trial that not even Kafka could have imagined, with scrutiny from dozens of review committees questioning the value of preventive therapy for breast cancer. Their concerns ranged from worry about liver cancer due to findings in rats (despite the fact that tamoxifen had been given to several million women for adjuvant treatment with no evidence of an increase), disbelief that a non-medically trained person could take a major role in developing such a trial, and a general reluctance to embrace the prospects of giving preventive medicine to apparently healthy women. Fortunately, I had a lot of support from a wide range of colleagues, which has been essential for this highly collaborative work. John Forbes from Australia and Tony Howell from Manchester have been with me from the beginning, and without them the work would not have been possible.

**Table 1:** Breast cancer prevention trials using tamoxifen

Trial (entry dates)	Population	Number randomised	Agents (vs placebo) and daily dose	Intended duration of treatment
Royal Marsden (1986–1996)	High-risk family history	2471	Tamoxifen 20 mg	5–8 Years
NSABP-P1 (1992–1997)	High-risk women > 1.6% 5 year risk	13,388	Tamoxifen 20 mg	5 Years
Italian (1992–1997)	Normal risk hysterectomy	5408	Tamoxifen 20 mg	5 Years
IBIS-I (1992–2001)	> 2-fold relative risk	7139	Tamoxifen 20 mg	5 Years
Adjuvant overview (1976–1995)	Women with ER + operable breast cancer in 11 trials	~ 15,000	Tamoxifen 20–40 mg with or without chemotherapy in both arms.	3 Years or more (average ~ 5 years)

*IBIS-I*, International Breast Cancer Intervention Study-1.

Adapted with permission from ref.^51^

**Fig. 2:**
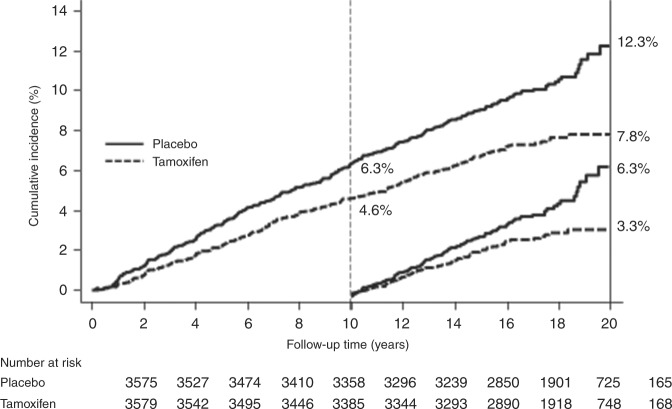
Long-term effect of tamoxifen on breast cancer prevention in the IBIS-I trial (modified from Cuzick et al.^15^)

Although the IBIS-I study was the first to begin recruitment, the NSABP-P1 trial was better funded and recruited more rapidly. As a consequence, they were able to report an initial positive result first,^9^ but in their desire to declare success at an early stage, long-term follow-up was not carried out in this study. By 2003, all four trials had shown a clear reduction in breast cancer risk,^10–14^ but the most exciting results were to come from the long-term follow-up of two of these studies. Both the Marsden study^15^ and IBIS-I^16^ continued long-term follow-up and the most recent report of IBIS-I, with a 16 year median follow-up, demonstrated that the 30% reduction in all breast cancer obtained from tamoxifen continued unabated till the end of follow-up, i.e., after treatment cessation at year five, the 30% reduction in new breast cancers continued for at least another 10–15 years (Fig. 2). The reasons for this have not been established at a mechanistic level, but a likely explanation is that tamoxifen is reversing early changes in the carcinogenic process, which are known to take at least 20 years, so that—at least in some cases—the process must start again for new tumours to develop. Breast cancer mortality reductions have yet to be seen, but this reflects the fact that even longer follow-up is required to see this,^17^ because at that time there were 503 breast cancers but only 57 deaths from breast cancer in the IBIS-I trial. Thus, another 10 years of follow-up will probably be needed to demonstrate a reduction in breast cancer mortality.

Subsequent to the tamoxifen prevention trials, clinical trials of three other selective oestrogen receptor modulators were conducted, primarily to look at the role of these agents in preventing fractures in women with osteoporosis. They all showed clear reductions in breast cancer and this subsequently became a second primary endpoint for the Continuing Outcomes Relevant to Evista (CORE) extension of the Multiple Outcomes of Raloxifene Evaluation (MORE) trial of raloxifene, in a subsequent follow-up period (Table 2

**Table 2:** Other SERMs that have been evaluated for effect on reducing breast cancer incidence in randomised trials

Trial (entry dates)	Population	Number randomised	Agents (vs placebo) and daily dose	Intended duration of treatment
MORE (1994–1999)	Normal risk, post-menopausal women with osteoporosis	7705	Raloxifene 60 or 120 mg (3 arm trial)	4 Years
CORE (2000–2004)	Normal risk, post-menopausal women with osteoporosis	4011	Raloxifene 60 mg	Additional 4 years after 4 years in MORE
RUTH (1998–2000)	Post menopausal women ≥ 55 years with CHD or risk factors	10,101	Raloxifene 60 mg	5 Years
STAR (2001 –2005)	High-risk post-menopausal women > 1.6% 5-year risk	19,747	Raloxifene 60 mg vs Tamoxifen (20 mg)	5 Years
PEARL (2001–2007)	Normal risk, post-menopausal women with osteoporosis	8556	Lasofoxifene 0.25 mg or 0.5 mg (3 arm)	5 Years
GENERATIONS (2005 – 2009)	Normal risk, post-menopausal women with osteoporosis	9354	Arzoxifene 20 mg	5 Years

*CORE* Continuing Outcomes Relevant to Evista, *MORE* Multiple Outcomes of Raloxifene Evaluation, *SERM* selective oestrogen receptor modulator, *STAR* Study of Tamoxifen and Raloxifene.

In the STAR trial, the comparator was tamoxifen and breast cancer incidence was the primary endpoint. In other listed trials, fracture prevention was the primary endpoint and the comparator was placebo. Adapted with permission from ref.^51^

). Only one trial has directly compared raloxifene with tamoxifen for cancer prevention.^18,19^ This was the Study of Tamoxifen and Raloxifene (STAR) trial, or NSABP-P2, in which almost 20,000 women were randomised between these two agents. Despite the indications that it might be more effective than tamoxifen, based on an indirect comparison from the MORE trial of raloxifene vs placebo with other trials of tamoxifen vs placebo, this direct comparison indicated that it was about 25% less effective, although the side-effect profile was more favourable. Both tamoxifen and raloxifene have now been approved for prevention by the Food and Drug Administration in the United States and recommended for prevention by National Institute for Health and Care Excellence (NICE) in the United Kingdom.

**Fig. 3:**
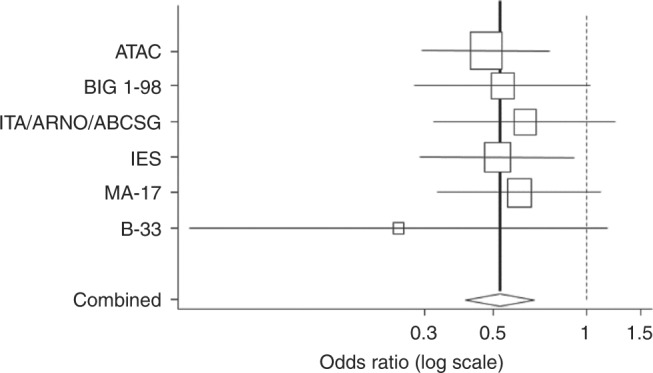
Reduced risk of contralateral breast cancer in trials comparing an aromatase inhibitor to tamoxifen. (Reprinted with permission from ref.^20^)

During this period, the Anastrozole Tamoxifen Alone or in Combination (ATAC) trial and other subsequent trials showed that aromatase inhibitors were more effective at reducing recurrence from breast cancer in the adjuvant setting than tamoxifen. I had the good fortune to be the statistician for the ATAC trial and I was able to monitor the data on contralateral tumours as they developed. It was with great excitement that I was able to show this data to the steering committee at time of the first planned efficacy analysis of the trial, which indicated that anastrozole was not only more effective than tamoxifen in preventing recurrence of breast cancer but was also more effective at preventing new disease in the contralateral breast. Other trials comparing tamoxifen with aromatase inhibitors subsequently also demonstrated this superiority^20^ (Fig. 3).

Thus, in 2003 we began the IBIS-II trial of anastrozole versus placebo in high-risk women. A major concern when planning this trial was whether the comparator should be placebo or tamoxifen and we had several long arguments about this. In the end, it was accepted that although tamoxifen clearly showed a reduction in cancer incidence, it was not widely used because of a lack of evidence for an overall long-term clinical benefit, and a full evaluation of side effects such as endometrial cancer, increased venous thromboses and a range of menopausal symptoms associated with oestrogen suppression was needed before this could be considered a “standard of care”. It was ultimately decided that the best design was to compare anastrozole to placebo. After a 5-year median follow-up, this trial showed a 53% reduction in all breast cancer,^21^ which was larger than the ~ 30% seen with tamoxifen in the four tamoxifen prevention trials. Another trial using the aromatase inhibitor exemestane also showed a strong reduction of 53% for all cancers and a 65% reduction for invasive cancers compared with placebo.^22^ Overall, the aromatase inhibitors are more active than tamoxifen, both for preventing disease recurrence and reducing the development of new cancers. An ongoing challenge, however, is to determine which individuals are more likely to respond to an aromatase inhibitor or tamoxifen, and today we still have few markers to guide that decision. The only current possibility is reduction in breast density at 6–12 months, which has been clearly established to be a predictor of response to tamoxifen^24–26^ and more recently appears to also be useful for aromatase inhibitors.^27,28^

One of the spin-offs from the prevention trials was the need to develop a model, which would predict the risk of breast cancer with sufficient accuracy to identify women at sufficient risk to be offered preventive tamoxifen or anastrozole. The model developed is known either as the IBIS model or the Tyrer–Cuzick model,^29^ reflecting my work with Jonathan Tyrer. Mathematically, it has several interesting features: in particular, it uses a segregation model to deal with family history of breast cancer, combined with a proportional hazards model to deal with the other known risk factors such as age, weight, reproductive history, hormone replacement therapy and prior benign breast disease. Subsequent versions of this model have been released and we are now using version 8, which was developed with Adam Brentnall. This includes a measurement of mammographic breast density and a single nucleotide polymorphism (SNP) score to look for low penetrance but common germline genetic differences.^30^

The model has now been widely validated and used to determine the need for preventive therapy or magnetic resonance imaging screening. More recently, it has emerged as a tool to guide the individual choice of screening intervals in so-called “risk adapted” screening algorithms. This may prove an important concept and would effectively expand screening programs to include a prevention component, with the idea being to conduct a risk assessment in all consenting women at their first screen. This would include a questionnaire to record “classical” factors as listed above, an assessment of density on their initial mammogram and a genetic SNP score based on a saliva sample. Several studies have now shown that these three factors are largely independent and contribute almost equally to the assessment of breast cancer risk^31–33^ (and several papers in preparation).

The biggest challenge now in therapeutic breast cancer prevention is not so much to identify high-risk women or specific effective agents, but to widely communicate this information in a way that is understandable to general practitioners and the general public.^34^ Uptake of preventive therapy has been very low^35^ and if it is to be an effective component of a comprehensive breast cancer prevention programme, e.g., as statins have been for the prevention of cardiovascular disease, we need to convince the medical profession and the public of the value of this approach.

## Use of low-dose aspirin for cancer prevention

Another area in therapeutic cancer prevention which has excited me greatly in recent years is the use of low-dose aspirin. A study more than 30 years ago by Waddell and Loughry^36^ showed that sulindac had a preventive effect on polyps in individuals with polyposis coli. Over the years, additional studies have shown the same is true for aspirin (Table 3

**Table 3:** Aspirin trials with colorectal adenoma as the primary endpoint

Study	Arm	Treatment duration (years)	Follow-up (years)	*N* (participants)	*N*(cases)	Relative risk (95% CI)
Baron et al.^52^	85 mg	3	3	377	140	0.81 (0.69–0.96)
	325 mg			366	160	0.96 (0.81–1.19)
	Placebo			372		
Sandler et al.^53^	325 mg	1	2.5	259	43	0.65 (0.46–0.91)
	Placebo			258	60	
APACC trial^54^	160/300 mg	4	5	126	38	0.73 (0.52–1.04)
	Placebo			112	46	

). A review of the available studies in 2009^37^ concluded that more evidence was needed to make a recommendation for widespread use, and further follow-up of ongoing trials was the most useful activity. Shortly after that study was published, further follow-up, led largely by Rothwell and colleagues^38^ in Oxford, began to emerge and provided strong evidence for a preventive effect on a range of cancers.

It is now apparent that aspirin has a large preventive effect on both the incidence and mortality from colorectal cancer, as well as gastric and oesophageal cancers.^40–42^ Small benefits are seen for lung, breast and prostate cancer in the order of 10%, but no other cancers appear to have been prevented by the use of aspirin (Table 4

**Table 4:** Benefits and harms of aspirin use that have been synthesised from more than 50 randomised trials and 100 epidemiologic cohort and case control studies

Event	Incidence	Mortality
Colorectal cancer	0.65	0.60
Oesophageal cancer	0.70	0.50
Gastric cancer	0.70	0.65
Lung cancer	0.95	0.85
Prostate cancer	0.90	0.85
Breast cancer	0.90	0.95
Myocardial infarction	0.82	0.95
Stroke	0.95	*1.21*
Major bleeding	*1.54*	–
GI bleeding	–	*1.60*
Peptic ulcer	–	*1.60*

GI, gastrointestinal.

**Fig. 4:**
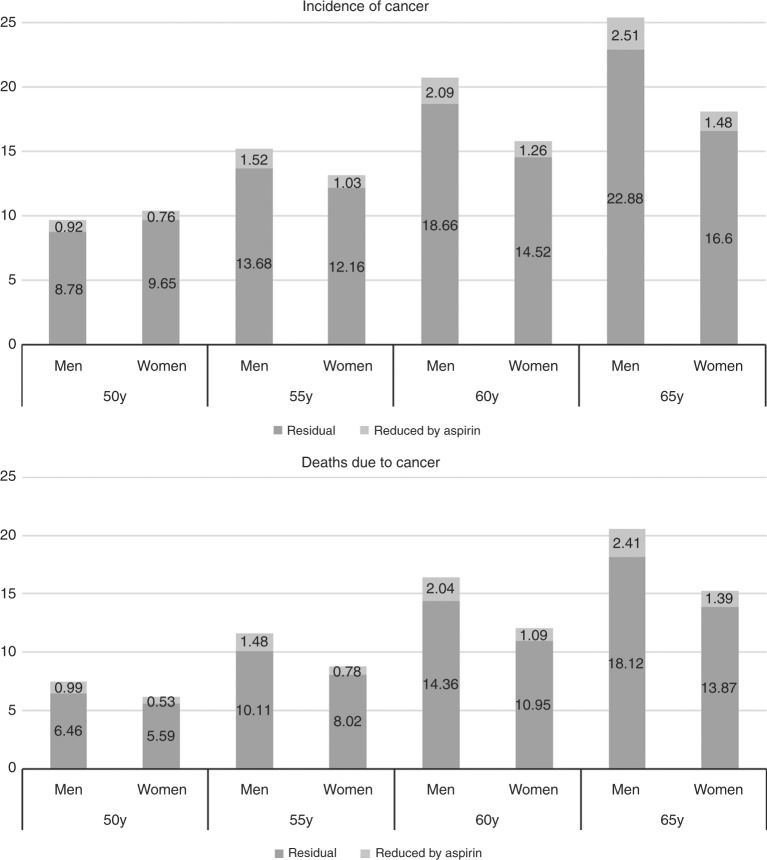
Estimated impact of use of aspirin for 10 years on the incidence and mortality of all causes of cancer (% of total population) by sex and age at starting. Estimates for incidence are for 15 years after starting aspirin and are 20 years after starting for mortality. Effect size is 7–10% reduction for incidence and 9–13% for mortality, depending on sex and age at starting. (Modified with permission from^39^)

Numbers are relative risks; those in italics indicate increased risk, all others indicate reduced risk. Adapted with permission from ref.^39,55^

). Overall, a reduction of around 10% is seen for all cancers combined if aspirin is taken for 10 years between ages 50 and 60 years, with a slightly larger impact on mortality (Fig. 4). These figures put aspirin second on a population basis, only below avoidance of tobacco as a cancer prevention approach, although this should not override the need for maintaining a good level of physical activity and avoiding obesity, as they are complementary and all have other major health benefits. Recommendations are now being made to offer aspirin for individuals at high risk of colorectal cancer,^43^ but a risk-benefit analysis from a group of experts strongly suggests that aspirin is suitable for a large proportion of the population,^39^ and that routine aspirin prophylaxis for at least 5 years should be offered to those between the ages of 50 and 70 years, if they have no contraindications based on risks of gastrointestinal (GI) bleeding or haemorrhagic stroke.^39^

Aspirin also shows promise for the adjuvant treatment of these cancers and a large trial in colorectal cancer is almost fully recruited (ASCOLT).^44^ A multisite trial (Add-Aspirin)^45^ in colorectal, gastric, oesophageal, breast, lung and prostate cancer is also now underway, as well as a smaller trial in early prostate cancer in combination with vitamin D (PROVENT).

The results on cancer prevention were a serendipitous finding in trials looking at low-dose aspirin to prevent cardiovascular disease, and as yet we have no clear understanding about the mechanism by which this takes place.^46^ Inflammation is likely to be involved in some way but the doses mostly used are too low for the standard Cox2 anti-inflammatory processes to be responsible; a major challenge is thus to understand more clearly the mechanisms by which aspirin prevents these cancers. We also need to more clearly understand who is at increased risk of GI bleeding, which is the major side-effect of aspirin even when used at low doses.

It is surprising that it took so long for the anti-cancer effects of aspirin to be discovered; a main part of the reason for this is that very little effect occurs in the first 5 years of follow-up after starting aspirin, and only long-term use for more than 5 years shows a beneficial effect on cancer, whereas the bleeding effects occur almost immediately. Thus, long-term follow-up is essential to determine the overall risk-benefit ratio of aspirin’s prophylactic use.

I believe that the evidence for an overall beneficial effect of aspirin is already strong enough to begin to make recommendations for the general population, after excluding those at high risk of bleeding. Nevertheless, several issues remain to help refine this indication. In particular the duration of use is not clearly established, although use for at least 5 years and probably 10 years is the minimum that should be offered. Continued use depends on the ratio of continued benefits versus increasing GI bleeding side-effects as an individual ages. Some evidence from the cardiovascular trials suggests a long-term benefit of aspirin after completion of the prescribed duration within the trial. However, it is not known how many of the participants continued to take aspirin after the trial was over and direct evidence on optimal duration of use is needed. Evidence on the risks of GI bleeding associated with aspirin in the elderly also needs refinement. A recent study indicated a clear increase in serious and fatal bleeds with aspirin in individuals aged more than 75 years, but it was shown that this could be avoided by the concomitant use of a proton pump inhibitor.^38^ It is not clear at which age a protein pump inhibitor should be offered, but its use does offer prospects for continuing prophylactic aspirin in older ages, which is certainly likely to be beneficial to cardiovascular disease prevention as well.

## HPV vaccination and treatment of precursor lesions

Another major advance in the area of ‘preventive therapy’ is the development of HPV vaccines. The newest nine-valent vaccine offers prospects of eliminating 90% of cervical cancer,^47,48^ together with an important proportion of other anogenital cancers as well as oropharyngeal cancer.

It also needs to be acknowledged that by identifying and treating precursor lesions, screening has had a preventive effect on cervix and colorectal cancers, and early detection has reduced the mortality from breast, prostate and lung cancer.

## Prospects for the future of therapeutic cancer prevention

Our successes to date in therapeutic cancer prevention have been based on repurposing drugs originally developed for other uses, i.e., endocrine agents used for breast cancer treatment, taking advantage of the fact that effects on the contralateral breast provide an opportunity to assess preventive effects. For aspirin, most of the trials were for cardiovascular disease prevention, and long-term follow-up provided clear evidence for an anti-cancer effect as well. Given the expense of running large prevention trials and developing new agents from scratch, it seems likely that future preventive agents will be identified from agents used for other indications and repurposed for cancer prevention. Programmes to actively explore opportunities to do this will be essential for expanding our portfolio of useful agents to prevent cancer. This is probably best achieved by mining general practice databases and large cohorts such as the UK Biobank. Promising agents include metformin, bisphosphonates, vitamin D and some dietary elements such as curcumin and sulforaphane.^49^

It is clear that a major obstacle in establishing the role of therapeutic cancer prevention is achieving wider acceptance by the profession and the general population. The cardiologists have been very successful in this respect; one aspect of their success has been to label risk factors such as high cholesterol or high blood pressure as diseases in their own right and thus worthy of treatment. Such factors are in short supply for cancer, and even in breast cancer, where risk assessment is the most developed, the only known marker is breast density (discussed above). We need to find ways to make preventive therapy more widely discussed and offered, and adding a prevention component to the breast cancer screening, and eventually other screening programmes, is one promising avenue. Aspirin has suffered from the fact that many authorities and societies that have made recommendations on preventive use reviewed it at a time before the benefits on cancer were known, and only  when considered for cardiovascular disease prevention alone, where the general population the risks associated with GI bleeding were similar to the benefits. This has changed dramatically with the discovery of a major effect on cancer incidence, which dominates any effect on cardiovascular disease, but sadly the older recommendations are still widely believed. One approach to establishing aspirin as a more widespread preventive agent would be to get NICE to review the evidence and hopefully make a recommendation for aspirin use in the general population.

Dr Sam Smith has made a major effort to understand the reluctance of general practitioners to recommend preventive therapy for cancer.^50^ His surveys have indicated that GPs are prepared to continue to prescribe preventive medicine such as tamoxifen or aromatase inhibitors to women at high risk of breast cancer, provided therapy is initiated in specialist centres and then referred to the general practitioner for continued use. This work and continuing studies in this area are essential if we are to achieve a change of opinion in the medical profession, which is key to widespread acceptance.^50^
